# Complicated Ulcerative Colitis in Pregnancy Due to Treatment Nonadherence: A Case Presenting As Suspected Appendicitis

**DOI:** 10.7759/cureus.92477

**Published:** 2025-09-16

**Authors:** Natalia Marisela Cabrera-Flores, Luis Adrian Alvarez-Lozada, Guillermo Elizondo-Riojas, Alejandro Quiroga-Garza

**Affiliations:** 1 Centro Universitario de Imagen Diagnóstica, Hospital Universitario "Dr. José Eleuterio González", Monterrey, MEX; 2 Human Anatomy, School of Medicine, Universidad Autónoma de Nuevo León, Monterrey, MEX; 3 General Surgery, Instituto Mexicano del Seguro Social, Monterrey, MEX

**Keywords:** appendicitis, clostridioides difficile infection, pregnancy-related complications, right hemicolectomy, ulcerative colitis

## Abstract

Ulcerative colitis (UC) is a chronic inflammatory disease of the colon that requires continuous treatment. Treatment nonadherence, as well as pregnancy, can complicate disease management. This case describes a 23-year-old primigravida at 23 weeks’ gestation with a history of UC who discontinued her treatment upon learning of her pregnancy. She presented with abdominal pain, bloody diarrhea, and systemic signs of infection. Imaging raised suspicion of appendicitis, and diagnostic laparoscopy revealed inflammatory involvement of the appendix and ascending colon, along with intra-abdominal fluid. Postoperatively, she was diagnosed with a severe UC flare and *Clostridioides difficile* infection. Despite antibiotic therapy, her condition progressed to fulminant colitis with bowel perforation, requiring an extended right hemicolectomy and ileostomy. Her course was further complicated by fetal demise, coagulopathy, aspiration pneumonia, hepatic congestion, and both arterial and venous thrombotic events, including an intracardiac thrombus. With multidisciplinary care, including anticoagulation and surgical wound management, she gradually improved and was discharged hemodynamically stable. This case highlights the high risk of adverse maternal and fetal outcomes when treatment is interrupted, the diagnostic complexity of the acute abdomen in this population, and the potential role of appendiceal involvement in disease severity.

## Introduction

Ulcerative colitis (UC) is a chronic inflammatory bowel disease (IBD) characterized by continuous inflammation of the colonic mucosa, beginning in the rectum with a variable degree of proximal extension. Typically, the disease starts in the late teens or early adulthood; however, it also occurs in children, with incidence increasing in recent years [1]. At least 50% of patients with IBD are diagnosed by age 35, and the disease most often affects women during their peak reproductive years. IBD poses a particular challenge during pregnancy because the health of the mother and fetus must be considered [2].

The etiology of IBD remains partially unclear, but several risk factors are known. Studies have shown that genetic susceptibility, impaired intestinal permeability, and an inappropriate immune response to gut microbiota are involved [3]. Although the pathogenesis of UC has not been fully determined, an abnormal mucosal immune response plays a major role in its occurrence and pathophysiology [4].

UC is characterized by continuous and diffuse colonic inflammation extending proximally from the rectum. Appendiceal involvement of UC, sometimes termed ulcerative appendicitis, has been recognized in 48-86% of patients with distal UC. Patients with ulcerative appendicitis experience a more aggressive and relapsing disease course compared with those without appendiceal involvement. Although the human appendix is considered a vestigial remnant, many case-control studies suggest that patients with UC who have undergone prior appendectomy are rare, have a delayed onset of UC, a reduced need for immunomodulators and proctocolectomy, and a reduced relapse rate and extent of UC [4-6].

This report describes the case of a pregnant woman with a history of UC who experienced severe clinical deterioration in the second trimester after discontinuing her treatment. The case highlights the serious consequences of treatment nonadherence in this population, including fulminant colitis, *Clostridioides difficile* infection, multiple thrombotic events, and fetal demise. It also discusses the diagnostic challenge posed by suspected appendicitis and outlines a comprehensive imaging and management approach. This case report adheres to the CARE checklist for case reports [7].

## Case presentation

A 23-year-old primigravida at 23 weeks of gestation, with a three-year history of ulcerative colitis managed with mesalazine, methylprednisolone, and metronidazole, presented with poor treatment adherence. She reported discontinuing all medications upon confirmation of pregnancy. She had also been hospitalized for pyelonephritis one month prior, treated with antibiotics, but did not receive follow-up.

Her current condition began seven days before admission, characterized by lumbar pain rated 9/10 in intensity, transfixing, and persistent, accompanied by 10 episodes of bloody diarrhea and fever. In the following days, the abdominal pain progressively worsened and was associated with nausea, diaphoresis, and hyperthermia. The symptoms were unresponsive to initial medical management, prompting her transfer to a tertiary hospital.

**Figure 1:**
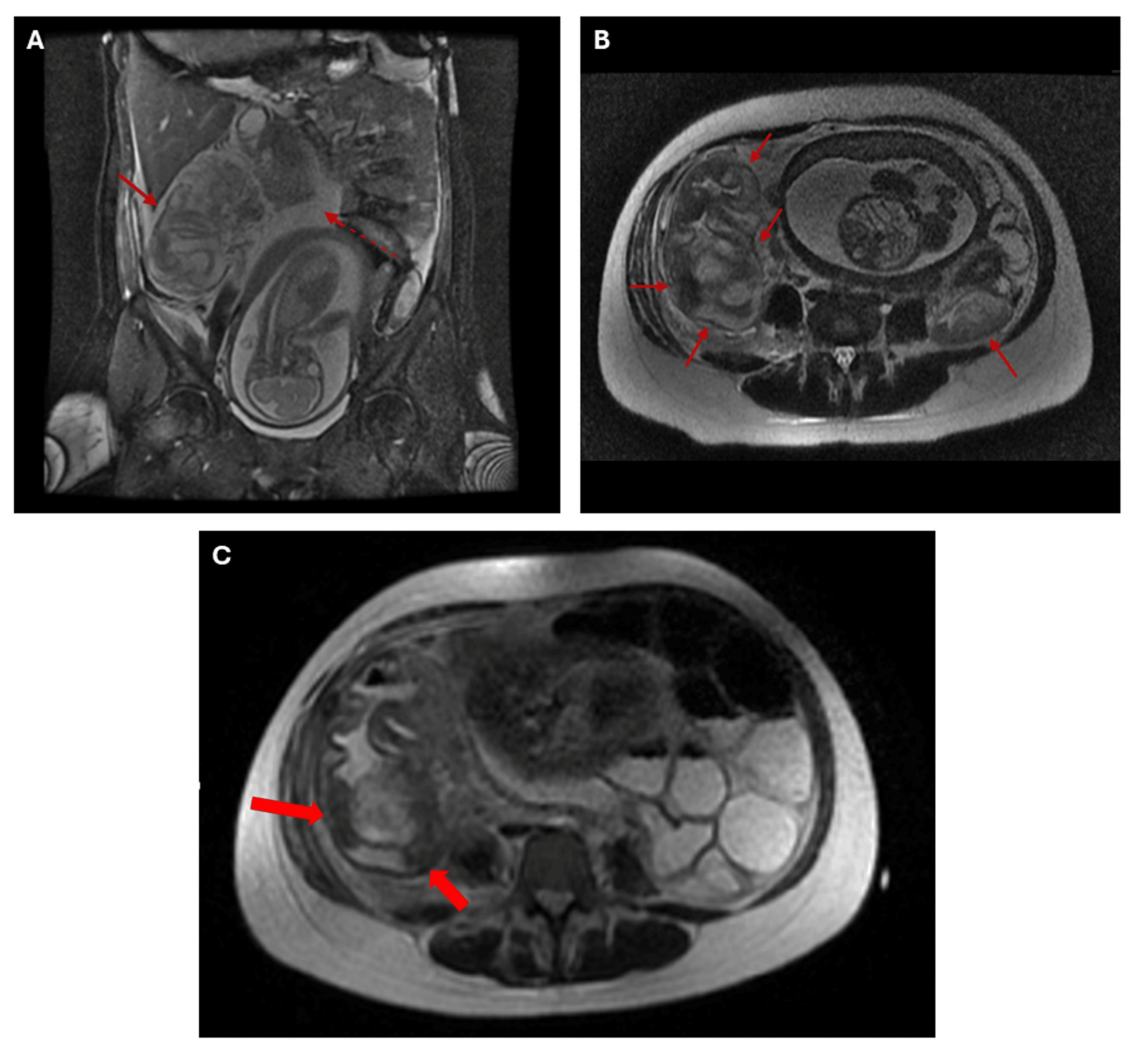
Coronal and axial abdominal MRI. A. Coronal FIESTA FS: Concentric, regular thickening of the submucosa of the cecum and ascending colon with areas of hypointense T2 signal (red arrows), suggestive of colitis of probable infectious versus inflammatory etiology, with associated submucosal hemorrhage. Free fluid is also noted (red dashed arrow). B. Axial T2 SSFSE: Concentric, regular thickening of the submucosa of the cecum and ascending colon with areas of hypointense T2 signal (red arrows), suggestive of colitis of probable infectious versus inflammatory etiology, with associated submucosal hemorrhage. C. Follow-up axial T2 SSFSE: Ascending colon with concentric wall thickening and low signal intensity of the submucosa, suggestive of blood content (microhemorrhages) (red arrows). FIESTA FS: Fast Imaging Employing Steady-State Acquisition with Fat Suppression; T2: T2-weighted sequence (spin-spin relaxation time); SSFSE: Single-Shot Fast Spin Echo.

Upon admission, her vital signs were 90/60 mmHg, 110 bpm, 20 rpm, 36 °C, and 98% SatO2. Consultations with internal medicine and general surgery were requested due to suspected acute abdomen, with appendicitis as the primary differential diagnosis. Abdominal ultrasound revealed findings suggestive of appendicitis, prompting further evaluation with abdominal MRI. The MRI demonstrated a cecal appendix with an enlarged diameter of 9.6 mm, along with free fluid in the hepatorenal recess, right paracolic gutter, right iliac fossa, and pelvis. Additionally, submucosal thickening of the cecum and ascending colon, measuring up to 12.0 mm and appearing reactive in nature, was noted (Figures 1A-1B).

Based on the clinical presentation and imaging, a diagnosis of secondary appendicitis due to ulcerative colitis exacerbation was established, and a decision was made to proceed with diagnostic laparoscopy. The procedure was performed the same day after the imaging studies.

During surgery, the ascending colon was found to have inflammatory infiltrative tissue and multiple adhesions to the abdominal wall, omentum, intestinal loops, liver, and gallbladder. The transverse colon, descending colon, and sigmoid colon were unremarkable. Approximately 700 cc of serohematic inflammatory fluid was identified within the abdominal cavity.

The initial postoperative course appeared uneventful for the first 48 hours; however, the patient was referred to gastroenterology due to signs of ulcerative colitis exacerbation two days post-surgery. Colonoscopy was performed, and *Clostridioides difficile* toxins A and B tested positive. Colonoscopic findings included fibrin blood clots and friable, erythematous mucosa with loss of vascular pattern. Three days post-surgery, the patient developed new episodes of severe abdominal pain (8/10), hemoglobin drop, diaphoresis, tachycardia, and hypotension, prompting consultation with critical care. She was admitted to the ICU with the diagnosis of fulminant *Clostridioides difficile* infection. Monitoring, hemodynamic support, and antibiotic therapy with vancomycin and metronidazole were initiated. A follow-up MRI performed the same day revealed bilateral pleural effusion, free abdominal fluid, and inflammation of the terminal ileum, cecum, ascending colon, and proximal transverse colon, with submucosal edema and microhemorrhagic zones in the ascending colon (Figure 1C).

Two days after the follow-up MRI, an extended right hemicolectomy with diverting ileostomy was performed due to imaging findings of intestinal ischemia and suspected perforation. Prior to the procedure, fetal viability was confirmed by the perinatology team. A midline laparotomy was carried out, and upon entering the abdominal cavity, approximately 150 cc of hemoperitoneum was observed. Intraoperative exploration revealed a gravid uterus without abnormalities, dilated intestinal loops, and signs of ischemia affecting the ascending colon and hepatic flexure. A 1 cm sealed perforation was identified in the ascending colon. A 50 cm segment of distal ileum was resected along with the ischemic colon. A transverse colonic staple line (greca) was created using 2-0 Prolene, consistent with a Hartmann-type procedure. A loop ileostomy was fashioned and exteriorized. The abdominal cavity was irrigated with copious sterile solution, and a Blake drain was placed in the surgical field. The fascia was closed using a running suture with 1-0 Prolene, the ileostomy matured with 3-0 Prolene, and the skin was closed with surgical staples. Fetal cardiac activity was again confirmed at the conclusion of the procedure by the perinatology team. The perforation prompted the addition of tigecycline to the patient’s treatment regimen.

Postoperatively in the ICU, the patient required invasive mechanical ventilation. Bronchoaspiration occurred during surgery, necessitating bronchoscopy, which did not reveal any aspirated content within the airways. Bronchoalveolar lavage was performed, and samples were sent for culture.

Gynecology and perinatology post-surgical evaluation confirmed the absence of fetal heart activity, and ultrasound verified fetal demise. Labor induction was initiated the next day using intravaginal misoprostol, followed by delivery. During hospitalization, the patient experienced high ileostomy output, managed with loperamide and IV fluid replacement. *Clostridioides difficile* toxin assays from ileostomy output were negative. Treatment with rectal vancomycin and metronidazole was continued.

**Figure 2:**
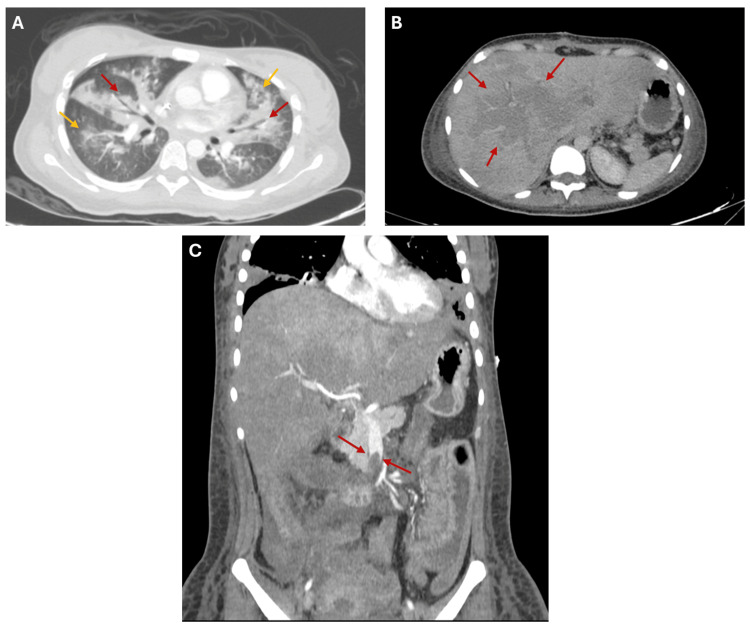
Chest and abdominal computed tomographies. A. Contrast-enhanced chest computed tomography: Bronchovascular thickening (red arrows) and peribronchial consolidation (yellow arrows), suggestive of pulmonary edema. B. Contrast-enhanced abdominal computed tomography: Perfusion abnormality of the liver compatible with “nutmeg liver” (hepatic venous congestion) secondary to systemic thromboembolic disease (red arrows). C. Contrast-enhanced abdominal computed tomography in the arterial phase: Thrombosis of the superior mesenteric artery (red arrows).

While in the ICU, one day after delivery, the patient developed prolonged coagulation times, raising suspicion of disseminated intravascular coagulation (DIC). Pulmonary embolism was suspected, and chest computed tomography angiography revealed aspiration pneumonia, hepatic congestion, and thrombosis of the superior mesenteric artery (Figure 2).

Management included transfusion of four units of plasma and one unit of packed RBCs. Further evaluation revealed thrombosis in the left internal jugular and subclavian veins, as well as an intracavitary thrombus in the left ventricle, prompting optimization of anticoagulation with enoxaparin.

After 16 days, the patient was discharged from the ICU and transferred to the internal medicine ward. She was conscious, maintaining normal oxygen saturation on room air, experiencing minimal abdominal pain, and exhibiting normal ileostomy output. She remained afebrile while transitioning from enoxaparin to warfarin.

The patient remained stable during her nine-day hospitalization until she developed surgical wound bleeding attributed to warfarin use. IV vitamin K was administered, and the wound remained open with mild bleeding for three days. Two days later, general surgery closed the wound, and anticoagulation therapy was continued post-intervention. The patient remained stable, and after eleven days, warfarin was reinitiated as bridging therapy with enoxaparin. Three days later, no further bleeding was observed. She remained hemodynamically stable, tolerated oral intake, ambulated, and remained afebrile for four consecutive days, leading to her discharge with close outpatient follow-up.

## Discussion

Pregnancy induces anatomical and physiological changes that may alter the presentation and management of gastrointestinal diseases. IBD, particularly UC, complicates pregnancy due to its relapsing nature, the risks associated with medication discontinuation, and the need to balance maternal and fetal outcomes. In this case, the patient’s decision to discontinue treatment early in pregnancy was a likely trigger for a severe disease flare, consistent with studies showing that non-adherence in pregnant UC patients significantly increases the risk of relapse and adverse outcomes [8].

The diagnosis of acute abdomen during pregnancy is especially challenging. Appendicitis, the most common non-obstetric surgical emergency in pregnancy, may present atypically and can overlap with IBD flares [9]. Imaging findings in this patient suggested appendicitis but also revealed colonic inflammation and hemorrhagic features indicative of UC exacerbation, aligning with prior reports that highlight the diagnostic overlap between appendicitis and IBD in pregnant patients [10]. Furthermore, the presence of appendiceal involvement in UC, also referred to as ulcerative appendicitis, has been associated with a more severe disease course and higher colectomy rates [4, 10].

In our patient, the use of MRI was crucial in clarifying the etiology of her acute abdomen while minimizing fetal risk. MRI has been described as a reliable, noninvasive modality for evaluating disease activity in UC, with sensitivity reported between 87 and 100% and specificity between 82 and 100% compared with endoscopy or histopathology [11, 12]. Importantly, MRI can also detect transmural inflammation and extraintestinal complications that may not be evident with endoscopy, which is especially relevant in pregnant patients where invasive procedures may be limited [11]. In the context of suspected appendicitis, MRI has similarly demonstrated high diagnostic accuracy, with sensitivities up to 96% and specificities up to 93%, and in pregnant women specifically, sensitivities of 50-75% with specificity approaching 100% [13, 14]. In this case, MRI provided essential findings suggestive of appendicitis while also revealing colonic inflammatory changes, guiding the decision for surgical exploration. These advantages underscore MRI as a safe and effective diagnostic modality in pregnant patients with IBD presenting with acute abdominal symptoms, balancing maternal and fetal considerations while informing timely therapeutic strategies.

*C. difficile *infection, as seen in this case, is a known complication of IBD, with pregnancy adding immunologic vulnerability. Fulminant colitis with perforation is rare but represents a critical emergency often requiring surgical management, as supported by ECCO and clinical guidelines [15]. Our patient underwent an extended right hemicolectomy with ileostomy, a procedure that has been associated with increased fetal morbidity in similar scenarios [9, 15]. Deeb M et al. described a comparable case in which delayed recognition of acute severe UC during pregnancy led to colonic perforation, emergency colectomy, and preterm delivery [16]. Likewise, Quddus A et al. reported a third-trimester patient who developed toxic megacolon requiring urgent surgical intervention despite medical therapy [17]. These cases, like ours, highlight the importance of early escalation to surgical management when conservative treatment fails, particularly when fetal viability must be weighed against maternal survival.

Thromboembolic events are a well-documented risk in both pregnancy and active IBD. In this patient, multiple thromboses developed, including involvement of the superior mesenteric artery, jugular vein, subclavian vein, and even an intracardiac thrombus, illustrating the additive risk of untreated inflammation and hypercoagulability [15]. Current guidelines recommend thromboprophylaxis in hospitalized IBD patients, and this case underscores its importance.

This case illustrates the complex intersection between UC, pregnancy, and acute abdominal presentations. The absence of preventive strategies due to treatment non-adherence, compounded by overlapping diagnoses and delayed intervention, underscores the need for proactive rather than solely reactive management. Preconception counseling offers a critical opportunity to align reproductive planning with disease control, ensuring that patients enter pregnancy in remission and thereby reducing the likelihood of severe, multi-organ complications such as those described here. Equally important is shared decision-making during pregnancy, as concerns about medication safety may prompt discontinuation of therapy. This highlights the necessity of clear, evidence-based discussions that balance the risks of untreated disease against those of pharmacologic treatment. As shown in this case, treatment interruption can precipitate catastrophic sequelae, including fulminant colitis, opportunistic infection, thromboembolic events, and fetal loss. Ultimately, proactive counseling, adherence to therapy, and coordinated multidisciplinary management before and during pregnancy are essential to safeguard both maternal and fetal outcomes.

## Conclusions

This case underscores the high risk of adverse maternal and fetal outcomes when ulcerative colitis is left untreated during pregnancy. The patient’s decision to discontinue therapy, driven by concerns about medication safety, resulted in a severe flare complicated by *Clostridioides difficile* infection, bowel perforation, multiple thromboembolic events, and ultimately fetal demise. Our report illustrates the serious maternal and fetal consequences of treatment interruption in ulcerative colitis during pregnancy. It highlights the importance of achieving disease control prior to conception, maintaining adherence to therapy, and engaging in shared decision-making informed by evidence on medication safety. Early counseling and coordinated multidisciplinary management are essential to prevent misdiagnosis, reduce the risk of catastrophic complications, and optimize outcomes for both mother and child.
